# *Neospora* spp. Seroprevalence and Risk Factors for Seropositivity in Apparently Healthy Horses and Pregnant Mares

**DOI:** 10.3390/ani12192699

**Published:** 2022-10-07

**Authors:** Lea Mimoun, Amir Steinman, Ynon Kliachko, Sharon Tirosh-Levy, Gili Schvartz, Elena Blinder, Gad Baneth, Monica Leszkowicz Mazuz

**Affiliations:** 1 Division of Parasitology, Kimron Veterinary Institute, Beit Dagan 50200, Israel; 2 Koret School of Veterinary Medicine, The Robert H. Smith Faculty of Agriculture, Food and Environment, The Hebrew University of Jerusalem, Rehovot 7610001, Israel

**Keywords:** *Neospora*, horses, seroprevalence, risk factors, neosporosis

## Abstract

**Simple Summary:**

Neosporosis, caused by the parasite *Neospora* species, is recognized as one of the major causes of abortion in cattle worldwide causing large economic losses. Over the past few years, *Neospora* infection and parasite transmission from the mare to the fetus has been documented in horses and was associated with reproduction failure. In the present study, we investigated parasite prevalence and associated risk factors in the general equine population and in a group of pregnant mares during pregnancy and after parturition. Our findings revealed high exposure of horses to *Neospora* spp. parasites, with significantly higher prevalence in pregnant mares.

**Abstract:**

Equine *Neospora* infection has been linked to neurological disorders and infertility in horses. This study looked into the risk factors for infection and the exposure to *Neospora* spp. in horses. The study was performed in two independent populations in Israel. The first consisted of apparently healthy horses, and the second consisted of mares examined during pregnancy and after parturition. Sera samples collected from horses and mares were tested for *Neospora* exposure by the indirect fluorescent antibody test (IFAT). The study revealed seroprevalence of 24% in apparently healthy horses and 66.4% and 48.6% in mares during gestation and after parturition, respectively. Among the investigated risk factors, older age (*p* = 0.026) and housing in both stalls and paddocks (*p* = 0.033) in apparently healthy horses, and Arabian breeds (*p* = 0.005) in pregnant mares, were found to be significantly associated with *Neospora* spp. seropositivity in univariable, but not multivariable, statistical analysis. This study revealed high exposure of equines to *Neospora* parasites, especially mares. Horse farm management, in combination with active surveillance, including serological testing and follow up, could help reduce the spread of the parasite among horses in endemic areas.

## 1. Introduction

Neosporosis is considered a serious disease of cattle and dogs worldwide. The disease, caused by the protozoan parasite *Neospora caninum*, is recognized as one of the major causes of abortion in cattle, leading to large economic losses in the livestock industry. *Neospora* is an obligatory intracellular parasite belonging to the phylum Apicomplexa, with dogs and related canids as definitive hosts [1]. *Neospora caninum* have been reported to infect various host species [2], while *N. hughesi* has only been reported in horses (and its definitive host has yet to be recognized). In the past, both *N. caninum* and *N. hughesi* have been reported in horses, and were associated with abortions and neurological disorders, respectively [3,4].

*Neospora* infection in intermediate hosts is frequently asymptomatic, and exposure is confirmed by the presence of antibodies against *Neospora*. Detection of anti-*Neospora* antibodies in serum samples of mammals is the most frequently used method in the study of the epidemiology and prevalence of neosporosis in live animals [5]. However, the potential of cross-reaction between *N. caninum* and *N. hughesi* in serological tests complicates the interpretation of the results of serological surveys, as serological differentiation between these two organisms is probably not possible [6]. Therefore, previous studies in horses based solely on serology cannot be considered species-specific.

The seroprevalence of *Neospora* spp. in horses has been reported in many regions. Antibodies against *Neospora* were found in 2% of equine serum samples in South Korea [7], 2.5% in South America [8], 11.5% in North America [9], 23% in France [10] and 23.8% in New Zealand [11]. A serological survey performed on horses in Israel (2007) showed a seroprevalence for *Neospora* spp. of 11.9% among 800 apparently healthy horses. A significantly higher seropositivity was found in horses with neurological signs (21.2%) and in aborting mares (37.5%) [3]. A recent study from Israel identified *Neospora* infection in aborting mares and aborted fetuses. All *Neospora* infections found in aborted fetuses were classified as *N. caninum* by molecular analysis [12]. *Neospora hughesi* has not been identified in horses in Israel, so far.

*Neospora caninum* is one of the most efficiently transplacentally transmitted parasites [13]. Transplacental transmission occurs when tachyzoites from the mare cross the placenta and infect the fetus. Fetal damage may occur due to tissue damage caused by the multiplication of *N. caninum* in the fetus or in the placenta [14]. A survey conducted in Brazil demonstrated the importance of transplacental transmission in horses. Among 129 seropositive mares, 34.8% gave birth to positive pre-colostral foals. The seroprevalence in mares was higher than in foals and seropositive mares were likely to transmit the parasite to their offspring, demonstrating that *Neospora* can be disseminated via the placenta [15]. A study performed in California over a two-year period, which followed 74 paired samples of mares and foals, showed that *Neospora* spp. persists in the equine population via endogenous transplacental infection [16]. In this study, three foals had pre-colostral antibody titers ranging from 2560 to 20,480 for *Neospora* spp. An additional study confirmed neosporosis as the cause of abortion in an equine fetus aborted after 280 days of gestation by histology, immunohistochemistry, ultrastructure and molecular analysis [17]. A recent study demonstrated high seroprevalence for *Neospora* in a group of aborting mares in Israel (70.9% among 31 mares). Positive titers varied from 1:50 to 1:6400, and 13 (out of 31) fetuses were found to be positive for *N. caninum* by polymerase chain reaction (PCR) [12].

In this study, we investigated the exposure to *Neospora* spp. in two horse populations. The first population consisted of apparently healthy horses from different areas of Israel. The second population was comprised of a group of mares examined during pregnancy and after parturition. Seroprevalence of *Neospora* spp. was examined in both groups to better understand the risk factors associated with seropositivity and the dynamics of antibodies in mares during and following pregnancy.

## 2. Materials and Methods

### 2.1. Experiment Design

The survey was conducted on two independent study populations. The first study population consisted of a group of apparently healthy horses selected to represent the geographical distribution of the Israeli equine population. In this group, horses were sampled once. The second study population was comprised of pregnant mares, sampled on two occasions, during gestation (mainly in the first trimester) and after parturition. In some instances, serum collection postpartum could not be performed due to lack of owner consent, change in ownership, or mare death. Horse details, including age, sex (for asymptomatic horses’ group), breed, reproduction history (for the mares’ group), geographic location, housing management (stalls, paddocks, pasture) and presence of dogs in the farm, were recorded and used for further analyses of possible risk factors for seropositivity. Sample collections were performed with the horse owners’ consent, and the study was approved by the Internal Research Committee of the Koret School of Veterinary Medicine—Veterinary Teaching Hospital (KSVM-VTH/02_2018, HU-NER-2020-055-A).

### 2.2. Study Population

Samples were obtained from two independent study populations: 

First population: Blood samples from 334 horses were collected during 2018–2019 from 30 farms, representing the geographical distribution of the Israeli equine population. Four to thirty-three horses were sampled at each farm. The farms were mainly riding schools, therapeutic riding schools and provided trail riding for inexperience riders. Few of the horses were sport horses (reining, jumping or dressage), and one farm kept endurance horses. Most of the horses were born in Israel; several had been imported, but not recently. This population was comprised of 161 mares (48.2%), 165 geldings (49.4%) and 8 stallions (2.4%) of various breeds. Horses’ age ranged from six months up to 47 years (mean (M) = 11.7; standard deviation (SD) = 6.04). 

Second population: Blood samples from 152 pregnant mares were collected at 36 farms located in different geographical locations in Israel during 2019. One to twenty sera were sampled at each farm. A total of 107 serum samples after parturition (in some cases after abortion or fetal absorption) were collected. The mares’ age ranged from two to twenty years old (M = 7.6; SD= 3.4).

### 2.3. Anti-Neospora spp. Antibodies Detection by Indirect Fluorescent Antibody Test (IFAT)

Blood samples were collected from each horse’s jugular vein into sterile vacuum tubes without anticoagulant. Blood samples were centrifuged at 2500 × rpm for six min. Sera were collected and samples were kept frozen at −20 °C until being processed. All samples were examined for the presence of antibodies against *Neospora* spp. by IFAT, using culture-derived NC-1 tachyzoites prepared in-house, as previously described [3]. In brief, serum samples from the general equine population were tested at 1:50 and 1:200 dilutions. Serum samples from the pregnant mares were tested at an initial screening dilution of 1:50. All samples that showed fluorescence at the initial dilution were considered positive, and were further diluted at a 1:2 ratio to the endpoint titer. Positive and negative controls were included on each slide. The highest dilution of serum exhibiting fluorescence of the whole *Neospora* organism was considered as the endpoint titer.

### 2.4. Statistical Analysis

The data were analyzed for association between demographic, environmental and husbandry parameters, and *Neospora* seropositivity. Fisher’s exact or Chi-squared tests were used for categorical variables, as appropriate, and odds ratios (OR) were calculated. Student’s *t* test and Spearman’s correlation were used for continuous parameters (age). All factors that were found to be significantly associated with *Neospora* seropositivity were included in a multivariable generalized estimating equation (GEE) using the logit link function, with the horse defined as subject and the farm as within-subject effect. Statistical significance was set at *p* < 0.05. The analysis was performed using the SPSS v.25 (IBM corporation, 2017) and WinPepi (J.H. Abramson, 2016) statistical softwares.

## 3. Results

### 3.1. Exposure to Neospora spp. in the First Study Population (Apparently Healthy Horses)

#### 3.1.1. Seroprevalence of *Neospora* spp.

**Figure 1:**
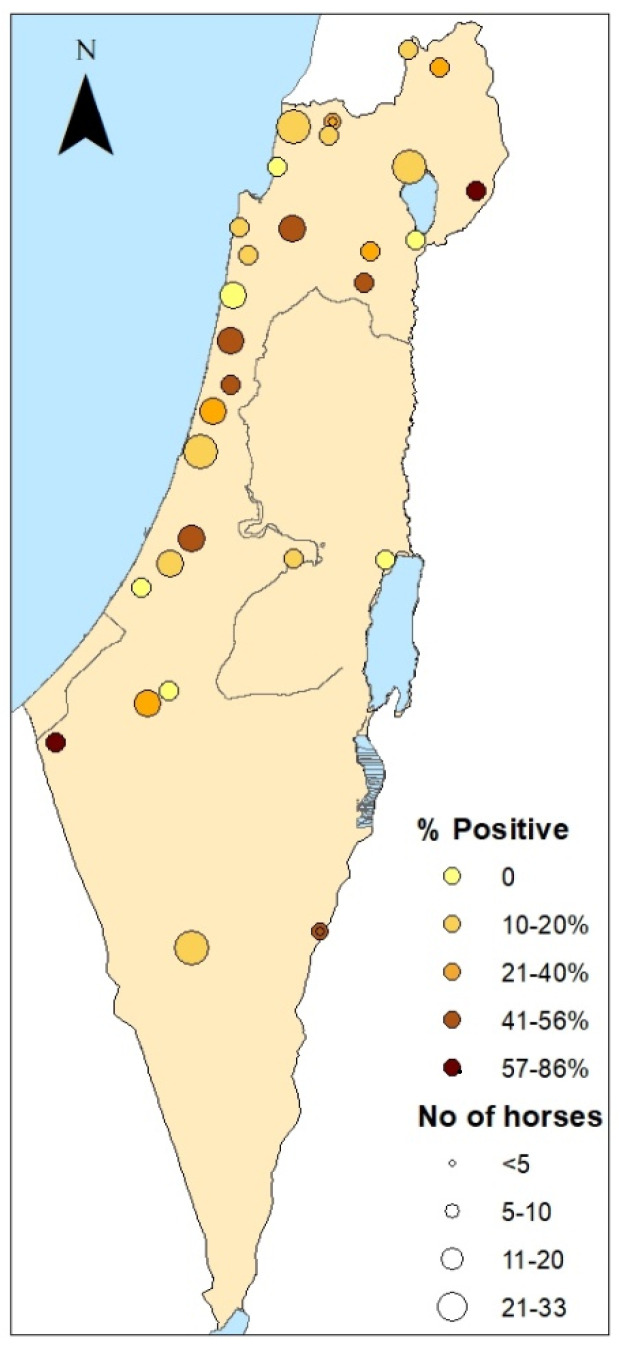
Map representing the geographical distribution of *Neospora* spp. exposure in horses in farms in Israel according to their geographical location. The seropositivity (using IFAT 1:50 cutoff-titer) to *Neospora* spp. at each farm is expressed by color intensity and the number of horses by the size of the circle.

The overall seroprevalence of *Neospora* spp. found in the general equine population was 24% (80 of 334). Of them, 6.6% (22 of 334) presented the titer of 1:200. Of the 30 farms examined, 24 (80%) were positive for *Neospora* spp., with an intra-farm seroprevalence ranging from 10% to 86% (Figure 1). High seroprevalence was found in the Golan Heights and Western Negev areas (57% and 86% respectively).

#### 3.1.2. Risks Factors Associated with *Neospora* spp. Infection

Associations between environmental and demographic factors and exposure to *Neospora* spp. using the 1:50 cut-off titer are summarized in Table 1. Significant difference in seropositivity was found between horses with different housing management. Horses kept in both stalls and paddocks had significantly higher seroprevalence than horses kept in either stalls, paddocks, or pasture (OR = 2.12, 95% confidence interval (CI): 1.18–3.76, *p* = 0.008). Age was also associated with *Neospora* spp. seropositivity. Mean age of seropositive horses was significantly higher than that of seronegative horses (*p* = 0.026, Table 2), and a weak but significant correlation was found between horses’ age and serological status (rho = 0.134, *p* = 0.015). Neither age (*p* = 0.073) nor housing (*p* = 0.061) was found to be significant in the multivariable model. *Neospora* spp. infection was not found to be significantly associated with horse’s sex, breed or geographical area. None of the factors tested was found to be significantly associated with seropositivity, when using a 1:200 cutoff titer.

### 3.2. Neospora spp. Exposure in Mares during and after Pregnancy 

#### 3.2.1. Seroprevalence to *Neospora* spp. and Pregnancy Follow up

Among the 152 sampled pregnant mares, 66.4% (95% CI: 58.3–73.9%) were found seropositive for *Neospora* spp., with titers ranging from 1:50 to 1:400. Following parturition, the seroprevalence was significantly reduced to 48.6% (95% CI: 38.8–58.5%). The odds ratio for being seropositive was twice higher in mares during gestation than in mares after parturition (OR = 2.09; 95% CI: 1.22–3.59%, *p* = 0.005).

Data regarding the pregnancy outcome was available for 135 mares. Of them, 125 had a healthy foal and ten experienced problems during pregnancy or parturition. Three mares aborted between the sixth and seventh month, three absorbed and four had dystocia (two of them died during parturition). The three aborting mares were seropositive for *Neospora* spp. during and after parturition (Table 3). Of the mares that absorbed their fetuses, one was seropositive, and one was seronegative for *Neospora* spp. during gestation, however, post-partum serum could not be collected. The third mare which absorbed its fetus was negative during and after pregnancy. Of the mares with healthy foals, 68% (85 out of 125) were seropositive for *Neospora* spp. No abortions were observed among seronegative mares, apart for one absorption.

#### 3.2.2. Variations in Anti-Neospora Antibody Titers during and after Gestation

Paired data from mares during pregnancy and after parturition were obtained from 107 mares (Table 3). Antibody levels remained the same after parturition in 34.6% of the pregnant mares. Antibody levels increased in 22.4% and decreased in 43% of them. There were significantly higher rates of mares with decreased antibody titers postpartum than with increased antibody titer (OR = 2.61, 95% CI: 1.39–4.96%, *p* = 0.002). Seroconversion from negative to positive after parturition was found in 42.4% of the pregnant mares and seroconversion from positive to negative was found in 48.6% of them. These rates were not significantly different (*p* = 0.411).

#### 3.2.3. Risk Factors Associated with *Neospora* spp. Infection in Pregnant Mares

The differences in seropositivity based on housing, pregnancies in the past, abortion history, proximity to dogs or cattle, food conditions, food access by canids and geographical location were not significant in pregnant mares and mares after parturition. Seropositivity was found to be associated with Arabian breeds both during pregnancy and following parturition. The proportion of seropositive Arabian mares was significantly higher than in the other breeds (during pregnancy: OR = 3.51, 95% CI: 1.28–9.51%, *p* = 0.008; after parturition: OR = 4.32, 95% CI:1.24–17.05%, *p* = 0.016, Table 4). The mean age of seropositive mares after parturition was significantly lower than that of seronegative mares (*p* = 0.006). A significant negative correlation was found between age and seropositivity after parturition (rho = −0.285, *p* = 0.006). Neither breed (*p* = 0.062) nor age (*p* = 0.164) was found to be associated with *Neospora* spp. seropositivity after parturition in the multivariable analysis. A positive tendency was found for mares after parturition living in proximity to dogs in the farm (Table 4).

## 4. Discussion

The seroprevalence of *Neospora* spp. was higher in both study populations compared to the seroprevalence found in horses in 2007 in Israel [3]. The present study revealed seroprevalences of 24% and 66.4% in apparently healthy horses and in pregnant mares, respectively, compared to 11.9% and 37.5% in the survey conducted on apparently healthy horses and aborting mares in 2007. The increase in seroprevalence of *Neospora* spp. parasites in horses from 2007 to 2020 may suggest an expanded exposure to *Neospora* spp. over those years in Israel.

The risk factors associated with *Neospora* spp. infection in the univariable analysis were housing management and age for apparently healthy horses and breed and age for pregnant mares. However, none of these factors remained significant in the multivariable model. Previous studies from other geographical areas, including neighboring Jordan, which evaluated risk factors associated with neosporosis in horses also did not reveal any significant factors in the multivariable analyses [18,19,20,21].

A seropositive tendency was found for post-partum mares living near dogs where there is a possibility of oocyst contamination and horizontal transmission. Exposure to dogs was found to be a significant risk factor for *Neospora* spp. infection in cattle and other intermediate hosts in other studies [18,19,22,23]. Since canids are defined as the definitive hosts of *N. caninum* [13,24,25,26], we assume that horses living not only in stalls but also in paddocks or pasture are more likely to be in contact with oocysts shed by the definitive host and to be infected by horizontal transmission than horses living only in stalls.

The geographical area was not found to be associated with seropositivity in both study populations. In apparently healthy horses, the seroprevalence varied between farms, and the number of horses sampled at each location varied between four and 33 horses. Higher seroprevalence was found in two farms located in northern and southern regions in Israel (57% and 86%, respectively). However, the number of horses sampled at each farm was relatively low (between four and ten horses) compared to the other farms (between 11 to 33 horses). Overall, it was impossible to conclude whether some farms had greater risk of infection. It is interesting to note that, in a previous study performed in Israel, the geographic area was found to be a risk factor to *Neospora* spp. infection as mares from the north of Israel had lower exposure than mares from central and southern Israel (46.1% versus 88.9%, *p* = 0.017) [12]. However, the sample size in that study was relatively small.

Most of the previous studies which evaluated the association between various risk factors and seropositivity only performed univariable statistical analysis [3,18,20,26]. Several factors were identified as significantly associated with *Neospora* seropositivity, most of them related to the management of the farm and the horses. These include routine use of anthelmintics [21], proximity to other animal species [19,20], feeding management [19,20], the use of horses [19] and quarantine [19]. In the few studies which also performed multivariable analysis [19] and the current study none of these factors remained significantly associated with exposure. This may suggest that neosporosis is a multifactorial infection and that the risk of infection is related to different environmental and management parameters, while antibody titers of chronic carriers may vary according to the animal’s general health and reproductive status. Also, many of the management parameters are linked when horses are sampled at the same farm and the contribution of each individual factor is difficult to assess. Therefore, better understanding of the pathogenesis and host-parasite interactions is needed to comprehend the complex epidemiology of neosporosis.

The present study demonstrated that *Neospora* spp. seropositivity is higher in mares, with rates of 66.4% during pregnancy and 48.6% after parturition, than in the general equine population (24%). This difference cannot be attributed to differences in sex, as seropositivity did not differ between geldings, stallions, and mares. Most mares sampled as a part of the general horse population were not pregnant, but the reproduction status was not recorded in all cases. Nonetheless, when comparing the seroprevalence only in mares in the general population (24.2%) and pregnant mares (66.4%, OR = 6.2, 95% CI: 3.68–10.47, *p* < 0.001) or mares after parturition (48.6%, OR = 2.96, 95% CI: 1.7–5.16, *p* < 0.001), both these differences are statistically significant. In addition, it is relevant to point out that all three aborting mares in the pregnant group were found seropositive during and after gestation. These findings are in accordance with a study revealing high seropositivity of 70.9% (22 of 31) in mares after abortion using the same serological cut-off value 1:50 [12]. Mares during gestation are in a stressful period, which may affect the life cycle of *Neospora* and generate modifications to the immune system response, thus providing an opportunity for parasite recrudescence and multiplication [27]. 

Anti-*Neospora* antibody titer fluctuations were found in paired samples of mares. Only 34.6% of these mares examined had the same antibody titers during pregnancy and after parturition. A decline in antibody titer after parturition occurred more frequently than augmentation. Seropositivity in pregnant mares was significantly higher than in mares after parturition (*p* = 0.005). Bradyzoite cysts are the chronic stage of infection which can escape recognition by antibodies [27]. In pregnant or stressed animals, bradyzoites can reconvert into tachyzoite, emerge out of the cysts, and then be recognized by the immune system, leading to a potential fetal loss due to transplacental infection [27,28]. We assume that recognition of the parasite by the immune system leads to an augmentation of anti-*Neospora* antibodies in the mare’s blood, explaining the increased antibody levels founded during pregnancy. 

The pregnancy outcome was not found to be significantly associated either with an increase or a decrease in antibody titer.

## 5. Conclusions

Overall, findings from the two study populations revealed an increase in *Neospora* spp. exposure in the Israeli equine population over the past few years. Several risk factors were found to be associated with *Neospora* spp. infection, including belonging to the Arabian breeds, older age and housing in both stalls and paddocks. These findings, combined with a previous report demonstrating *Neospora* infection in aborted fetuses, highlight that the prevalence and clinical significance of neosporosis in horses is increasing. Horse farm management, in combination with active surveillance, including serological tests and follow up, could help to reduce the spread of neosporosis in horse farms in Israel. Moreover, long-term follow-up studies are required in order to determine the impact of parasite infection on mare fertility and on the development of neurological signs in horses.

## Figures and Tables

**Table 1:** *Neospora* spp. seroprevalence in the first study population of apparently healthy horses, according to the different risk factors (sex, breed, geographical location, and housing management).

Risk Factor		N	Cutoff Titer 1:50		Cutoff Titer 1:200	
			N Positive (%)	*p* Value	N Positive (%)	*p* Value
Sex	Mare	161	39 (24.2)	0.580	14 (8.7)	0.323
	Gelding	165	38 (23)		8 (4.8)	
	Stallion	8	3 (37.5)		0 (0)	
Breed	Mixed	152	39 (25.7)	0.107	9 (5.9)	0.657
	American	76	16 (21.1)		4 (5.3)	
	Pony	19	6 (31.6)		2 (10.5)	
	European	20	3 (15)		2 (10)	
	Draft	2	1 (33.3)		0 (0)	
	Arab	44	14 (31.8)		5 (11.4)	
	Gaited	13	0 (0)		0 (0)	
	Andalus	1	1 (100)		0 (0)	
Mixed vs. Pure-bred	Mixed	152	39 (25.7)	0.619	9 (5.9)	0.597
	Pure-bred	176	41 (23.3)		13 (7.4)	
Geographical area	North	157	37 (23.6)	0.645	9 (5.7)	0.544
	Center	88	24 (27.3)		8 (9.1)	
	South	89	19 (21.3)		5 (5.6)	
Housing	Stall	112	19 (17)	**0.033**	4 (3.6)	0.068
	Stall and paddock	86	30 (34.9)		11 (12.8)	
	Paddock	78	18 (23.1)		5 (6.4)	
	Pasture	58	13 (22.4)		2 (3.4)	

**Table 2:** *Neospora* spp. seroprevalence in the first study population of apparently healthy horses detected by IFAT in relation to horse’s age.

Horse Serology Titer for *Neospora* spp.	N (%) *	Horse Age Mean	Standard Error (SE)	*p* Value
Negative	250 (75.8)	11.33	0.67	
Positive (cut-off titer 1:50)	80 (24.2)	13.06	0.38	**0.026**
Positive (cut-off titer 1:200)	22 (6.7)	13.14	1.08	0.265
Total **	330	11.7	0.332	

* Total number of sera from horses analyzed by IFAT immunoassay. ** The age of four horses was not recorded.

**Table 3:** Anti-*Neospora* antibody titer variation detected by IFAT between mare’s pregnancy period and after parturition.

Mare Serum Antibody Titers		Number of Mares	Pregnancy Outcome *
During Pregnancy	After Parturition		
Negative	Negative	19	17 normal parturitions 1 dystocia 1 absorption
	1:50	6	normal parturition
	1:100	5	normal parturition
	1:200	3	normal parturition
1:50	Negative	21	normal parturition
	1:50	10	9 normal parturitions 1 abortion during the 6th month
	1:100	6	normal parturition
	1:200	2	normal parturition
1:100	Negative	12	normal parturition
	1:50	8	7 normal parturitions 1 abortion during the 7th month
	1:100	7	normal parturition
	1:200	2	normal parturition
1:200	Negative	3	normal parturition
	1:50	1	normal parturition
	1:100	1	1 abortion during the 10th month
	1:200	1	normal parturition
Total		107	

* Data are not shown for all the ten mares who had an abnormal pregnancy because sera could not be collected after pregnancy for all of them.

**Table 4:** *Neospora* spp. seroprevalence in mares during gestation and after parturition in relation to various descriptive factors.

Risk Factor *		*Neospora* spp. Seroprevalence in Pregnant Mares			*Neospora* spp. Seroprevalence in Mares after Parturition		
		*N1*	Positive (%)	*p_1_*	*N2*	Positive (%)	*p_2_*
Breed	Arab	81	64 (79.0)	**0.005**	61	37 (60.7)	**0.019**
	Mixed	7	3 (42.9)		6	2 (33.3)	
	Other, pure-bred	29	15 (51.7)		19	5 (26.3)	
Housing	Stall	57	39 (68.4)	0.616	40	22 (55.0)	0.264
	Paddock **	87	56(64.4)		64	28 (43.8)	
Pregnancies in the past	0	18	10 (55.6)	0.280	13	9 (69.2)	0.654
	1	18	14 (77.8)		12	6 (50.0)	
	2 or more	32	24 (75.0)		26	15 (57.7)	
Abortion history ***	No previous abortion	83	54 (65.1)	1.00	62	28 (45.2)	0.824
	Previous abortion	14	9 (64.3)		12	5 (41.7)	
Food storage condition	Open field	51	32 (62.7)	0.997	41	25 (61)	0.099
	Enclosed space	59	37 (62.7)		42	18 (42.9)	
Presence of cattle next to the farm	Yes	27	21 (77.8)	0.076	25	14 (56.0)	0.314
	No	98	58 (59.2)		70	31 (44.3)	
Presence of dogs in the farm	Yes	114	72 (63.2)	0.354	84	42 (50.0)	0.070
	No	16	12 (75)		13	3 (23.1)	
Food access by canids	Yes	68	40 (58.8)	0.187	55	30 (54.5)	0.188
	No	54	38 (70.4)		37	15 (40.5)	
Geographical location	North	33	24 (72.7)	0.635	28 56 23	14 (50.0)	0.622
	Center	91	58 (63.7)			25 (44.6)	
	South	28	19 (67.9)			13 (56.5)	
Total		152	101 (66.4)		107	52 (48.6)	

* Not all data was available for every mare. ** The term “paddock” includes mare living only in paddocks or both in stalls and paddocks. *** The abortion history also includes cases of absorption.

## Data Availability

The data presented in this study are available on request from the corresponding author.
